# Deep and comparative analysis of the mycelium and appressorium transcriptomes of *Magnaporthe grisea *using MPSS, RL-SAGE, and oligoarray methods

**DOI:** 10.1186/1471-2164-7-310

**Published:** 2006-12-08

**Authors:** Malali Gowda, RC Venu, Mohan B Raghupathy, Kan Nobuta, Huameng Li, Rod Wing, Eric Stahlberg, Sean Couglan, Christian D Haudenschild, Ralph Dean, Baek-Hie Nahm, Blake C Meyers, Guo-Liang Wang

**Affiliations:** 1 Department of Plant Pathology, Ohio State University, Columbus, OH 43210, USA; 2 Department of Plant and Soil Sciences, University of Delaware, DE 19711, USA; 3 Arizona Genomics Institute, Department of Plant Sciences, University of Arizona, Tucson, AZ 85721, USA; 4 Ohio Supercomputer Center, The Ohio State University, Columbus, OH 43210, USA; 5 Agilent Technologies Inc, Little Falls Site, 2850 Centerville Road, Delaware 19711, USA; 6 Solexa, Inc. 25861 Industrial Blvd, Hayward, CA, 94545, USA; 7 Fungal Genomics Laboratory, Department of Plant Pathology, North Carolina State University, Raleigh, NC 27606, USA; 8 Department of Biological Science, Myongji University, Kyonggido, 449728, Korea

## Abstract

**Background:**

Rice blast, caused by the fungal pathogen *Magnaporthe grisea*, is a devastating disease causing tremendous yield loss in rice production. The public availability of the complete genome sequence of *M. grisea *provides ample opportunities to understand the molecular mechanism of its pathogenesis on rice plants at the transcriptome level. To identify all the expressed genes encoded in the fungal genome, we have analyzed the mycelium and appressorium transcriptomes using massively parallel signature sequencing (MPSS), robust-long serial analysis of gene expression (RL-SAGE) and oligoarray methods.

**Results:**

The MPSS analyses identified 12,531 and 12,927 distinct significant tags from mycelia and appressoria, respectively, while the RL-SAGE analysis identified 16,580 distinct significant tags from the mycelial library. When matching these 12,531 mycelial and 12,927 appressorial significant tags to the annotated CDS, 500 bp upstream and 500 bp downstream of CDS, 6,735 unique genes in mycelia and 7,686 unique genes in appressoria were identified. A total of 7,135 mycelium-specific and 7,531 appressorium-specific significant MPSS tags were identified, which correspond to 2,088 and 1,784 annotated genes, respectively, when matching to the same set of reference sequences. Nearly 85% of the significant MPSS tags from mycelia and appressoria and 65% of the significant tags from the RL-SAGE mycelium library matched to the *M. grisea *genome. MPSS and RL-SAGE methods supported the expression of more than 9,000 genes, representing over 80% of the predicted genes in *M. grisea*. About 40% of the MPSS tags and 55% of the RL-SAGE tags represent novel transcripts since they had no matches in the existing *M. grisea *EST collections. Over 19% of the annotated genes were found to produce both sense and antisense tags in the protein-coding region. The oligoarray analysis identified the expression of 3,793 mycelium-specific and 4,652 appressorium-specific genes. A total of 2,430 mycelial genes and 1,886 appressorial genes were identified by both MPSS and oligoarray.

**Conclusion:**

The comprehensive and deep transcriptome analysis by MPSS and RL-SAGE methods identified many novel sense and antisense transcripts in the *M. grisea *genome at two important growth stages. The differentially expressed transcripts that were identified, especially those specifically expressed in appressoria, represent a genomic resource useful for gaining a better understanding of the molecular basis of *M. grisea *pathogenicity. Further analysis of the novel antisense transcripts will provide new insights into the regulation and function of these genes in fungal growth, development and pathogenesis in the host plants.

## Background

One of the most daunting challenges in the post-genomic era is to identify all transcribed regions in a sequenced genome. Although computational programs have played an important role in genome annotation, highly accurate prediction of the coding regions of many genes is still challenging [1]. Therefore, the experimental approaches such as ESTs (expressed sequenced tags) [2], full-length cDNA sequencing [3], SAGE (serial analysis of gene expression) [4,5], MPSS (massively parallel signature sequencing) [6,7], RATE (robust analysis of transcript ends [8] and microarrays [9,10] are essential tools to validate the annotated putative transcriptional units (TUs) in the sequenced genomes.

Although EST and full-length cDNA sequencing are effective approaches for gene discovery and expression profiling, neither is cost-effective nor comprehensive enough to isolate the majority of rare transcripts. Since the fabrication of most types of microarrays relies on sequences that are derived from ESTs, full-length cDNAs or computer program-predicted coding regions, many rare transcripts may be absent from these arrays. SAGE and MPSS are two powerful methods for genome-wide expression profiling and novel gene identification [4,5,7]. Both methods determine the sequence of short tags derived from a defined position at the 3' regions of expressed mRNAs in a cell. SAGE uses traditional sequencing technologies, while MPSS uses a novel bead-based hybridization procedure [6]. The main advantage of MPSS over SAGE is that over one million signatures can be obtained from a library in comparison to 100,000 to 200,000 tags in a SAGE library. However, SAGE method is technically simple and can be performed in most molecular laboratories, whereas MPSS involves many complex cloning steps and library construction and sequencing is only performed at Solexa, Inc. The newly developed 5'-RATE method simplifies tag cloning and sequencing procedures and should have a broad application for transcriptome analysis of complex genomes [8].

*Magnaporthe grisea*, the causal agent of rice blast disease, causes severe yield losses to cereals, including the economically important crops, such as rice, barley, wheat, and millets [11]. This fungus completes its life cycle by conidia landing on the leaf surface, conidia germination, appressorium formation, penetration, establishment and new conidia formation within seven to ten days after infection [11,12]. In addition to its economic importance, the recent release of the complete genome draft sequence [13] and characterization of several important avirulence/pathogenecity-related genes [14], makes *M. grisea *a model plant pathogen for studying host-pathogen interactions. Although 12,841 genes (Annotation release 5, [15]) are predicted in the genome of *M. grisea*, many of these genes have no experimental support. Identification and characterization of all expressed genes from the infectious (appressoria) and non-infectious (vegetative mycelia) structures are indispensable for understanding the mechanism of fungal pathogenesis and designing novel strategies to combat the disease.

The main objective of this study was to identify all expressed transcripts encoded in the *M. grisea *genome using MPSS, RL-SAGE and oligoarrays, and to compare the qualitative and quantitative measurements produced by these three methods. Using these three technologies, the *M. grisea *transcriptome in the vegetative mycelial stage and in the infectious appressorial stage was analyzed in depth, in which many novel sense and antisense transcripts and alternative splicing variants were discovered. We also compared the correlation coefficients of the gene expression patterns revealed by MPSS, RL-SAGE and oligoarray. Our results provide the most comprehensive analysis of the sense, antisense and alternative transcripts of a fungal genome published to date and represent a useful genomic resource for further detailed functional analysis of the expressed transcripts involved in *M. grisea *growth and pathogenesis.

## Results

### Characterization of mycelium and appressorium transcriptomes of *M. grisea *by MPSS and RL-SAGE methods

Using MPSS, a total of 1,326,720 transcript tags from mycelia and 1,459,146 transcript tags from appressoria were obtained. As per Meyers et al. [7], we filtered the total MPSS tags into two groups: significant (= 4 copies in either library) and non-significant (= 3 copies in both libraries) tags. After performing a clustering analysis with the total tags, 12,531 (61.7%) and 12,927(76.7%) significant tags and 7,768 (38.3%) and 3,500 (21.3%) non-significant tags were identified from mycelial and appressorial tissues, respectively (Table 1). Clustering analysis of the significant tags from both mycelium and appressorium MPSS libraries resulted in 20,062 distinct tags corresponding to 8,669 annotated genes. Using both significant and non-significant tags, a total of 9,467 unique genes were identified. Approximately, 37.5% (7,531) of the appressorial significant tags and 35.6% (7,135) of the mycelial significant tags were specific to each stage. When the tags matched to the annotated genes, 1,784 and 2,088 genes specific to appressorium and mycelium tissues, respectively, were identified [see Additional File 1]. These results show that the transcriptomes of appressorium and mycelium tissues are quite different.

An RL-SAGE library was constructed using the same RNA sample that was used for the construction of the mycelium MPSS library (Table 1). The average insert size of the RL-SAGE clones was approximately 1.1 kb. A total of 245,873 individual tags and 51,925 distinct tags were obtained from 7,292 sequence reads. Out of the total distinct tags, 16,580 (31.9%) were significant (= 2 copies) and 35,345 (68.1%) were non-significant (single copy) transcript tags.

### Matching analysis of the MPSS and RL-SAGE tags to *M. grisea *genomic and expressed sequences

We compared the MPSS and RL-SAGE data to both genomic and expressed sequences of *M. grisea *to identify the genes from which these tags were obtained. The whole genome sequence from the Broad Institute (Release 5.0 on January 2006 at [15]) and the ESTs from TIGR (Release 5.0 on April 28, 2004 [16]) and the COGEME database [17]) were used to match MPSS and RL-SAGE tags. The matching results are summarized below.

#### 1) MPSS tags

The matching rate of the significant tags to the genomic sequence from both libraries was about 85% (Table 2). In contrast, the matching rate to *M. grisea *ESTs for the significant tags from the mycelial (61.8%) and appressorial (51.2%) libraries was much lower than that to the genomic sequence. Similarly, 40 to 50% of the significant tags matched to the predicted coding sequences (CDS) [13]. These results suggest that a large proportion of significant MPSS tags (at least 50% of tags in both the libraries) were not present in the public EST collections and could be considered as novel transcripts. Given that 5–10% of the ESTs may have no matches in the genome sequence, we conservatively estimate that at least 40% of the significant MPSS tags represent previously unidentified transcripts. We also observed that ~ 25 to 32% of the significant MPSS tags from both the libraries matched to regions within 500 bp downstream of the 3' region of annotated genes and about 7% of the significant tags from both the libraries matched to regions within 500 bp upstream of the 5' region of annotated genes. About 10 to 15% of the significant tags located in the intergenic regions matched to the genomic sequence but not to the CDS, 500 bp downstream or 500 bp upstream sequences (Table 2). Combining the CDS, 500 bp upstream and 500 bp downstream of CDS, we identified 7,686 genes in appressoria and 6,735 genes in mycelia by MPSS. A total of 3,362 genes were commonly expressed in both appressoria and mycelia. While 1,784 and 2,088 genes were specifically expressed at the appressorial and mycelial stages, respectively.

#### 2) RL-SAGE tags

In comparison with the MPSS libraries, more significant tags were identified in the mycelial RL-SAGE library. About 65% of significant RL-SAGE tags matched the *M. grisea *genomic sequences (Table 2). Unlike the MPSS tags, only 31% (5,067) of the significant tags from the RL-SAGE library matched to the *M. grisea *EST sequences (TIGR) and a small portion (16%) of the tags matched to the CDS of the annotated genes (Table 2). We also observed that about 31% of the mycelial RL-SAGE significant tags matched to the 500 bp downstream of the 3' region of the annotated genes and about 6% of the significant tags matched within 500 bp upstream of the 5' region of the annotated genes (Table 2). The reason for the high percentage of the RL-SAGE tags located within the 500 bp downstream region might be due to more *NlaIII *sites (RL-SAGE) than *DpnII *sites (MPSS) in the 3' UTR region. Combining the tags hit to CDS, 500 bp upstream and 500 bp downstream regions, we identified 6,028 genes in mycelia by the RL-SAGE method.

#### 3) Identification of novel transcript tags in the MPSS and RL-SAGE libraries

A detailed analysis of the novel transcripts that did not match to any sequences in the ESTs and annotated genes was performed (Table 2). The percentage of the novel tags in the appressorium MPSS, mycelium MPSS, and mycelium RL-SAGE libraries was about 35%, 26%, and 35%, respectively [see Additional File 2]. A total of 3,339 genes in the RL-SAGE library identified by CDS and their 500 bp upstream and down stream regions did not match the ESTs either in TIGR or COGEME database. Similarly, 3,186 and 2,298 genes identified in the MPSS appressorial and mycelial library, respectively, also did not show any matches in TIGR or COGEME *M. grisea *ESTs. In the two MPSS libraries, about 67% and 76% of the non-significant appressorial and mycelial tags matched to the genome sequence, respectively (Table 2). Even among the tags that did not match the genome sequence, many of them were significant tags, which might be derived from the un-sequenced or intron-exon junctions in the genome. Some of them may be true transcripts encoded in the genome but could not be matched due to sequencing errors in the genome or in the EST, MPSS or RL-SAGE tag sequencing. To validate the MPSS and RL-SAGE results, two genes (MGG_04847.5 and MGG_0490.5) without any EST matches in the public databases were amplified and cloned (data not shown), demonstrating that the majority of the identified novel tags might be true transcripts.

### Antisense transcripts for the annotated genes of *M. grisea*

#### 1) Antisense transcript tags from the MPSS libraries

**Figure 1:**
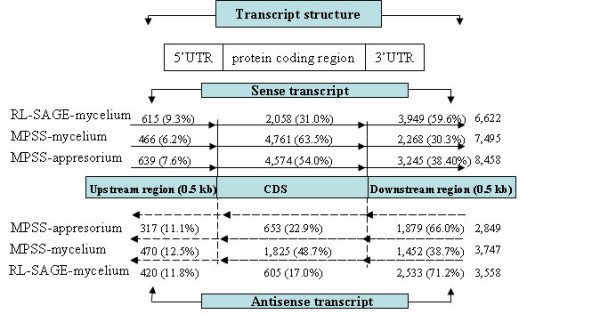
Location of MPSS and RL-SAGE tags on upstream (0.5 kb), coding sequence (CDS) and downstream (0.5 kb) regions of annotated genes of *M. grisea*. The sense and antisense tags locations are shown on the predicted transcription units of the *M. grisea *genes. The percentage of tags are shown within the parenthesis.

Numerous antisense tags were identified in this study that matched *M. grisea *annotated genes in an antisense orientation. A total of 3,747 significant antisense tags (1,825 from the coding region, 1,452 from the 500 bp downstream regions, and 470 from the 500 bp upstream regions) corresponding to 2,958 genes from the mycelial library and 2,849 significant antisense tags (653 from the coding region, 1,879 from the 500 bp downstream regions, and 317 from the 500 bp upstream regions) corresponding to 2,629 genes from the appressorial library were identified. Of which, 1,586 and 1,257 genes were mycelium and appressorium-specific, respectively. Among them, 1,372 antisense genes were commonly expressed in both appressoria and mycelia. Total antisense tags from significant and non-significant tags are shown in Figure 1. Only fewer significant antisense tags (653 tags) were identified in the appressorium library as compared to the mycelial library (1,825 tags). Interestingly, we observed antisense transcripts for some of the well-known genes that are involved in appressorium formation and pathogenesis such as hydrophobin (MPG1), and calmodulin [see Additional File 3]. When the tags only matching the antisense sequences and having a single hit to the annotated genes were chosen, 232 genes in appressoria and 274 genes in mycelia were identified as antisense transcripts. The antisense tags and their frequency are listed in Additional File 4.

#### 2) Antisense transcript tags from the RL-SAGE library

Although the total number of significant antisense transcript tags (3,558) corresponding to 3,100 genes identified from the mycelial RL-SAGE library was similar to that of the mycelial MPSS library, the tag distribution in the three regions of the annotated genes was different (Figure 1). The proportion of the RL-SAGE antisense tags located in the 500 bp downstream regions was twice (71.2%) that of the MPSS library (38.7%) at the mycelial stage. Similar with the sense tags, this difference might be due to the use of different anchoring enzymes in MPSS (*Dpn*II) and RL-SAGE (*Nla*III) library construction. Among the identified antisense tags, 364 were present in both RL-SAGE and MPSS libraries, 1,730 were specific to the MPSS libraries, and 431 were specific to the RL-SAGE library. The antisense tags and their frequency are listed in Additional File 4.

#### 3) Annotated CDS regions with sense and antisense MPSS tags

Nearly 19% of the annotated genes in the *M. grisea *genome had sense and antisense tags matching to both strands of the annotated genes. Among these genes, 2,075 (16% of the total annotated genes) were from the mycelium MPSS library and 774 (6.0%) were from the appressorium MPSS library. There were 1,627 genes with sense and antisense pairs specifically present in the mycelium library and 326 genes with sense and antisense pairs specifically present in the appressorium library. Four hundred and forty eight genes (3.5%) were present in the two libraries [see Additional File 5]. To support that the MPSS sense and antisense tags are reliable, we found that six annotated genes have both sense and antisense transcripts from the RL-SAGE library and public EST collections (Table 3).

#### 4) Annotated CDS regions with sense and antisense RL-SAGE tags

We identified that nearly 10% of the annotated genes in the *M. grisea *genome had bothsense and antisense tags from the mycelial RL-SAGE library(data not shown). The significant lower number of tag pairs at the protein coding region in the RL-SAGE library is likely because that the majority of the RL-SAGE tags were located in the 500 bp downstream region of the annotated genes (Figure 1). Some of the sense and antisense RL-SAGE tag pairs are also present in the MPSS libraries and public EST collections (Table 3).

### Identification of alternative transcript tags in mycelia and appressoria

Since MPSS and RL-SAGE tags are derived from the 3' end of each transcript, the presence of more than one tag in the CDS of a gene suggests an alternative termination of the gene. These termination differences could be derived from either alternative polyadenylation or alternative splicing at the 3' end. All the transcripts from both cases are called alternative transcripts in this study. To determine the extent of transcriptional diversity in *M. grisea*, we assessed the proportion of the genes with alternative terminations in both libraries.

#### 1) Alternative sense and antisense MPSS tags in mycelia and appressoria

When the sense tags were matched to the annotated genes, ~ 20–35% of the genes were found to have at least two alternative transcripts (Table 4). In general, more genes expressed in mycelia had more alternative transcript tags in comparison to that in appressoria. The hypothetical protein similar to linoleate diol synthase precursor (MGG_13239.5) in the mycelium MPSS library matched to 29 alternative tags and another hypothetical gene (MGG_01625.5) in the appressorium library had 12 alternative tags (data not shown). A total of 2,542 mycelial and 1,517 appressorial genes were found to encode alternative transcripts. Among them, 911 genes were common between the two tissues, 1,631 were mycelia-specific and 606 were appressoria-specific. Further, we used *M. grisea *TIGR EST database from which alternative splice forms from *M. grisea *were clustered. The genes undergoing alternative termination or splicing have more than one MPSS tags. A total of 47 appressorial and 55 mycelial unique clusters have two or more MPSS tags [see Additional File 6].

Similarly, many annotated genes had more than one antisense tags in the MPSS libraries (Table 4). Twenty percent of the annotated genes in appressoria and 32% in mycelia had more than one antisense tag. As with the sense transcript tags in mycelia, the same gene (MGG_13239.5) had thirteen alternative antisense tags (data not shown).

#### 2) Alternative sense and antisense RL-SAGE tags in mycelia

About one-fourth (27.5%) of the annotated genes in mycelia were found to produce at least two alternative sense tags in the RL-SAGE library, which is less than that observed in the MPSS mycelial library (Table 4). In the RL-SAGE mycelial library, a hypothetical protein similar to reverse transcriptase (MGG_13890.5) was found to encode sixteen alternative transcript tags (data not shown). Many genes with known functions were found to encode alternative transcript tags [see Additional File 7]. A total of 10,629 alternative tags were commonly present in both RL-SAGE and MPSS [see Additional File 8].

Among the antisense RL-SAGE tags in the mycelium library, nearly a quarter (24%) had at least two alternative antisense transcript tags per gene (Table 4). A hypothetical gene (MGG_00329.5) was found to generate seven alternative antisense transcript tags (data not shown). Several genes with multiple sense and antisense alternative tags were also identified. For example, four sense and one antisense tags were obtained for the HSP70 gene (data not shown).

### Characterization of the appressorium and mycelium transcriptomes by oligoarray hybridizations

To compare the transcriptional profiles generated from MPSS and RL-SAGE with that from oligoarray analysis, the same RNA samples used in MPSS and RL-SAGE library construction were hybridized to the *M. grisea/O. sativa *oligoarray [18]. Using a stringent cut off at false discovery rate (FDR) = 0.05 that corresponds to a *p-*value of 0.001, 9,138 genes (43.9%) were identified to be statistically significant expressed in mycelium and appressorium tissues. Among them, 8,569 probes are from *M. grisea *genes and 569 probes from rice genes. The hybridizations with the rice genes were likely due to sequence similarity between housekeeping genes in both organisms. Of the 8,569 *M. grisea *genes, 4,652 (54%) and 3,917 (46%) were differentially (2 fold) up-regulated in appressoria, and down-regulated in mycelia, respectively. We identified 846 *M. grisea *genes that were ≥ 3.0 fold significantly up-regulated in appressoria, and 792 genes that were ≤ 3.0 fold significantly down-regulated in mycelia. The top 20 highly and specifically up- and down-regulated genes in appressoria and mycelia are shown in [see Additional File 9].

**Figure 2:**
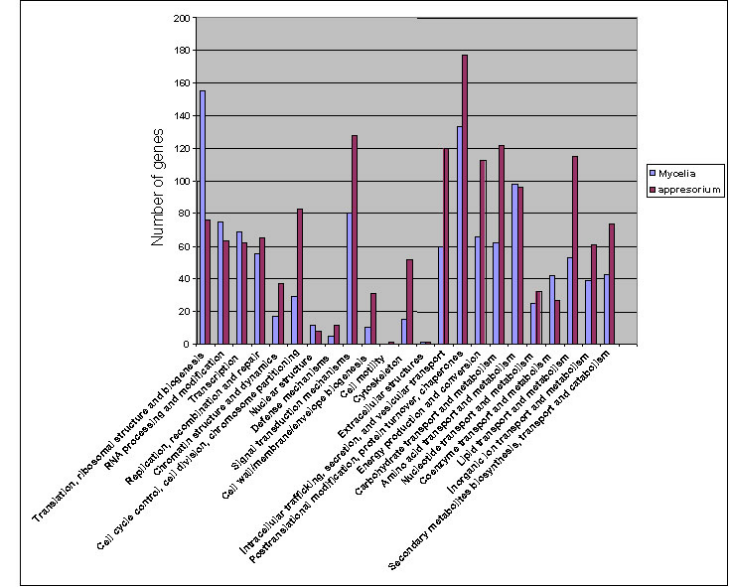
Abundance of the genes involved in different pathways in appressoria and mycelia. A total of 4,649 appressorial and 3,784 mycelial genes identified by microarray analysis were used in the KOG analysis.

To gain more insight on the molecular mechanisms involved in *M. grisea *pathogenesis, we tried to functionally categorize 8,569 significant expressed genes, that were induced or repressed in the appressorium (4,652 genes) and also in the mycelium (3,917 genes), respectively, into different functional classes using KOGs analysis based on putative function of proteins [19]. Functional classification and percentage of genes represented in appressorial and mycelial tissues are shown in the Additional File 10 and Additional File 11. The results indicate that a significant proportion of the genes (58% in appressoria and 64% in mycelia) were unclassified. The relative categories of genes expressed at the mycelial and appressorial stages are shown in Figure 2. The abundance of the gene category of cell cycle control, cell division, chromosome partitioning, cytoskeleton, lipid transport, and metabolism was significantly high in appressoria as compared to mycelia (Figure 2). On the contrary, the abundance of the genes involved in translation, ribosomal structure and biogenesis, RNA processing and modification, nuclear structure, coenzyme transport and metabolism, was significantly expressed in the mycelia when compared to appressoria.

### Qualitative analysis of the *M. grisea *transcriptome using MPSS, RL-SAGE, and oligoarray platforms

**Figure 3:**
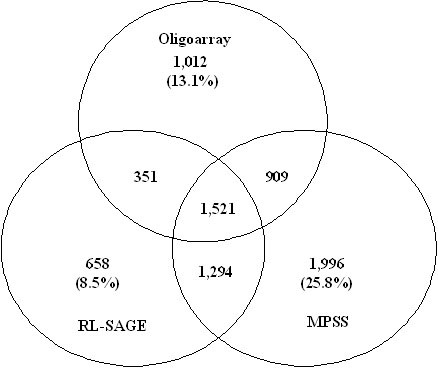
Venn diagram showing qualitative clustering analysis of *M. grisea *transcriptomes revealed by MPSS, RL-SAGE and oligoarray methods. A total of 7,741 unique genes which were present in all three platforms were used in the analyses. Three method comparison (A) and pair comparison (B) were performed. Only single-hit MPSS and RL-SAGE tags were used to match the sequences on the oligoarray.

To compare the expression pattern of the transcripts identified from the three different methods, a comparative analysis was performed. Because of the differences in the three platforms, a direct comparison of gene expression was not feasible. Therefore, an indirect comparison was made in which the oligoarray sequences (13,666) were used as the common targets to match the mycelial MPSS and RL-SAGE tags. The appressorium tags were not included in the analysis since no appressorial RL-SAGE library was made. Using the mycelial RL-SAGE (51,925) and MPSS (20,299) tags, 5,720 MPSS and 3,824 RL-SAGE tags matched the gene sequences on the microarray (13,660). When the expression profiles of the mycelial and appressorial tissues were compared, 3,793 genes were significantly expressed in the mycelial tissue. Clustering analysis of the genes from the three groups resulted in the identification of 7,741 genes. A total of 1,521 genes were commonly expressed in all three platforms (Figure 3). Oligoarray and RL-SAGE shared 32.58%, oligoarray and MPSS shared 34.30% and MPSS and RL-SAGE shared about 41.83% of the 7,741 genes. Oligoarray and MPSS together identified 91.49%, oligoarray and RL-SAGE together identified 74.21% and RL-SAGE and MPSS together identified 86.92% of the 7,741 unique genes. These results demonstrated that each method could identify specific groups of expressed genes and a combination of either of the two methods can identify the majority of the transcripts.

### Quantitative analysis of the mycelium and appressorium transcriptomes using MPSS, RL-SAGE, and oligoarray platforms

To quantitatively assess the transcripts identified in the three different platforms, a Pearson correlation coefficient analysis was performed. In a comparative analysis between MPSS or RL-SAGE mycelial tags and the oligoarray mycelial mean signal intensity, a poor correlation was observed when unfiltered data was used (Table 5). In the MPSS and oligoarray data comparison, regression analysis was performed after removing the ten genes that were within the regression standard residual cutoff of = -1.75 to = 5.3. The removal of this small number of outliers had increased the correlation co-efficiency from 0.18 to 0.51 (Table 5). Similarly, after removal of fourteen outlying genes (based on a residual cutoff of = -1.75 to = 5.3), the correlation coefficiency between RL-SAGE tag frequency and the oligoarray mean signal intensity was improved significantly (Table 5). For example, the correlation coefficiency was increased from 0.29 to 0.64 when RL-SAGE tags with copy number = 10 were used. In general, the correlation coefficiency was higher in the comparisons for the genes with high copy numbers.

Conversely, a moderate correlation coefficiency (0.59) was obtained between MPSS and the oligoarray appressorial data without data filtering (Table 5). After the removal of the four outlying genes, the correlation coefficiency was increased from 0.65 to 0.74 for the MPSS tags with copy number = 10 (Table 5). In mycelia, the correlation was only about 0.5 for the genes with 25 or more copies, even after the removal of ten outlying genes. Comparing mycelial MPSS and RL-SAGE tags data, a low correlation was observed without data filtering. After the removal of the 18 outliers from the dataset, the correlation coefficiency was increased from 0.068 to 0.4 between mycelial MPSS and RL-SAGE tags (Table 5).

In summary, we found a low to moderate correlation among the expression data from the three platforms, especially those data between MPSS and oligoarray. In general, a better expression correlation was observed for high copy number tags in the MPSS and RL-SAGE libraries with their corresponding genes on the oligoarray.

## Discussion

Recent technological innovations have advanced genomics in an unprecedented way. Several complex genomes have been sequenced in recent years providing an excellent starting point to fully understand the genetic blueprint of an organism. However, identification of all the expressed portions of a sequenced genome is a challenging task, yet critical to the understanding of gene regulation and metabolic networks. The public availability of the whole genome sequence of *M. grisea *has established a solid foundation to further understand the pathogenicity mechanism of this notorious fungal plant pathogen which causes severe yield losses in rice growing countries [13]. Elucidating the transcriptome of *M. grisea *may ultimately lead to the development of novel approaches for combating rice blast disease. In the last few years, many researchers have adopted various gene expression profiling techniques to characterize the *M. grisea *transcriptome under various conditions or in different cell types including EST sequencing [20], microarrays [13], and SAGE [21]. However, these methods have only provided partial information about the *M. grisea *transcriptome due to technical limitations and the depth of the surveys performed in these studies. In this study, we employed three global and quantitative expression tools, namely MPSS, RL-SAGE and oligoarrays, to profile the *M. grisea *transcriptome at two developmental stages. In these experiments, the same RNA samples isolated from mycelia and appressoria were used so that the results from three different platforms could be readily compared. A total of 12,531 and 16,580 significant tags in mycelia have been identified by MPSS and RL-SAGE, respectively. In appressoria, 12,927 significant MPSS tags were identified. Many identified transcripts were not present in the existing EST or cDNA collections of *M. grisea *and many of them matched unannotated regions of the genome.

Both RL-SAGE and MPSS are tag-based approaches for transcriptome analysis and genome annotation. They are different from the conventional approach that focuses on the large clone collections following the principle of collecting a "representative clone" for each gene. Although conventional approaches are useful to catalog the expressed genes in certain tissues, especially moderately or highly expressed genes, many weakly expressed genes might have been missed in these collections. More importantly, it is not possible for the conventional approaches to address the questions regarding the dynamics of transcriptional regulation and regulatory principles like alternative promoter usage and splicing [22]. In contrast, RL-SAGE and MPSS methods isolate and sequence short tags (17–21 bp) from the 3' regions of most transcripts. At least 100,000 RL-SAGE tags or a million MPSS tags can be easily obtained from these libraries.

In this study, we obtained approximately 1.3 and 1.4 million tags from the mycelial and appressorial MPSS libraries, respectively. The matching rate of the significant tags from each library to the *M. grisea *draft genome sequence was about 85%, suggesting that the MPSS data have a very deep coverage of the transcriptome. In contrast, only about 50 to 60% of the significant tags matched to the existing EST collections in the public databases. A similar result was also obtained from the RL-SAGE library, suggesting that current *M. grisea *EST collections are incomplete. One possible explanation is that most *M. grisea *ESTs were sequenced from the 5' region of the transcripts [23], whereas most of the MPSS and RL-SAGE tags were derived from the 3' region of expressed genes. Due to sequencing cost limitations, we only sequenced only 7,000 clones and obtained a quarter million tags from the mycelium RL-SAGE library. The transcripts recovered from the MPSS and RL-SAGE methods were overlapping but not identical due to the use of different anchoring enzymes in the library construction. Interestingly, we found that the genome matching rate of RL-SAGE tags is lower than that of the MPSS tags. These are two possible reasons. First, sequencing errors might generate unmatched tags, especially for singleton tags. Second, the significant MPSS tags used for matching have = 4 copies whereas the significant RL-SAGE tags have = 2 copies, suggesting the MPSS tags selected for matching may be more reliable. The last reason is that most of the RL-SAGE tags matched putative 3' UTR region, which may frequently targeted for RNA variation as reported in mammalian system [24]. Nevertheless, our results demonstrated that MPSS and RL-SAGE methods are powerful techniques for deep transcriptome analysis and novel gene discovery. The two methods are complementary and different types of transcripts could be identified from each of these methods.

One of the advantages of tag-based techniques is the detection of alternatively terminated transcripts in the RNA population. From both MPSS and RL-SAGE libraries, we found many annotated genes have alternative transcript tags. Some of them have corresponding EST transcripts. The percentage of genes with evidence of alternative termination ranged from 27% in the RL-SAGE mycelium library to 35% in the MPSS mycelium library. The higher rate of alternative transcript tags in the MPSS library may be due to the fact that more MPSS tags (66%) matched to the coding regions of the annotated genes than that of the RL-SAGE tags (37%). It has been previously shown that a high rate of alternative transcripts was found in the protein coding regions (74%), and a low rate (4%) of alternative transcripts was found in the 3' UTR [25]. The reason for the lower rate of alternative transcripts in appressoria than in mycelia is not clear. In addition, cloning and sequencing confirmation of some alternative transcripts without ETS support is required. Regulation of the alternative transcripts and functions of these sense and antisense alternative transcripts in *M. grisea *warrant more detailed analyses.

Naturally occurring antisense transcripts were first observed in prokaryotes and viruses and later found in eukaryotes. There is evidence for the involvement of antisense transcripts in alternative transcription [26,27], RNA editing [28,29], DNA methylation [30,31], genomic imprinting [32,33], and X-chromosome inactivation [34]. In this study, many antisense tags were identified in mycelium or appressorium libraries, corresponding to more than 25% of the annotated genes. It is not clear at this point that why M. grisea genome encode so many antisense transcripts. Like the sense tags, almost two-thirds of antisense transcripts detected in the RL-SAGE library were located in the 500 bp downstream regions, whereas the majority of antisense tags from the MPSS library were located within the protein coding regions. Surprisingly, we found that almost one-fifth of the annotated genes encode both sense and antisense transcripts. In these genes, antisense transcripts could form double stranded RNA (dsRNA) with their sense transcripts. If a dsRNA is formed, it could be degraded to form small interfering RNAs that could decrease sense RNA abundance [35]. Alternatively, interference by RNA polymerase II transcription activity on the antisense strand could restrict sense-strand transcription [36].

Recently, few studies demonstrated the function of antisense transcripts in fungal growth and development. For example, the circadian clock gene in the fungus *Neurospora*, a close relative of *M. grisea*, is regulated by the presence of natural antisense transcripts [37,38]. Casas-Flores et al [39] expressed an antisense version of the pkr-1 gene of *Trichoderma atroviride*, encoding the regulatory subunit of protein kinase A (PKA), resulted in a non-sporulating phenotype.

Through data mining for EST, MPSS and RL-SAGE antisense tags, we have identified longer antisense transcripts in *M. grisea *for transcription factors MST12 (MGG_12958.5) and DEAD box-containing protein (MGG_12894.5), ribosomal protein, S9 (MGG_12892.5), ribosomal protein, L34 (MGG_05296.5) and ATP synthase alpha chain (MGG_07752.5). MST12 (a yeast homologs of STE12) is essential for host penetration and invasive growth, but was not required for appressorium formation [40,41]. MST12 is regulated by MAP kinase at the downstream of signal transduction cascade during pathogenesis [40,41]. It will be interesting to know if the MST12-mediated signal transduction cascade is regulated by antisense mechanism or not. A detailed characterization of MST12 antisense transcript may reveal its novel role in pathogenesis.

MPSS, SAGE, and oligoarrays are three widely used methods for transcriptome profiling. We performed qualitative and quantitative comparative analysis of the mycelial and appressorial transcriptomes revealed by the three methods. More than 40% of the annotated genes were detected by both MPSS and RL-SAGE methods. There was a good correlation in gene expression levels between the appressorium MPSS expression data and appressorium oligoarray data (0.67) and a moderate correlation between the mycelium MPSS and the mycelium oligoarray data (0.49) after removing several outlying genes in the datasets. However, the correlation between RL-SAGE and MPSS or oligoarray data was not significant. The low correlation between RL-SAGE and oligoarray might be because that oligoarray probes are designed from the protein coding regions of the annotated genes and a large number of RL-SAGE tags are located in the 500 bp downstream regions (putative 3' UTRs). We speculate that a low correlation between MPSS and RL-SAGE is due to the following two reasons. First, the use of different anchoring enzymes would change the location of the MPSS and RL-SAGE tags within a given transcript. We found that the majority of the MPSS tags matched the protein coding regions and the majority of RL-SAGE tags matched within 500 bp downstream (putative 3' UTRs). In some cases, because the *M. grisea *annotation is incomplete, the MPSS and RL-SAGE tags derived from the same transcript may be mapped to two different predicted genes. Second, MPSS and RL-SAGE use two completely different library construction and sequencing procedures. Any bias in the PCR amplification of synthesized cDNAs could lead to generation of different tag populations. Nonetheless, these data provide the first detailed analysis of transcriptional activity in an important fungal pathogen of plants, and constitutes a starting point for large-scale functional analysis of many novel fungal genes identified in the study.

## Conclusion

We sequenced one RL-SAGE library of mycelia and two MPSS libraries of appressoria and mycelia of *M. grisea*. Using the same RNA samples of appressoria and mycelia, oligoarray hybridization was performed to check if these three approaches can detect similar sets of expressed genes in *M. grisea*. The distinct transcripts detected by MPSS and RL-SAGE in appressoria and mycelia ranged from 12,000 to 16,000, which correspond to about 9,000 genes, representing 80% of the predicted genes in *M. grisea *[13]. A low to moderate correlation among the expression data from the three platforms was observed. MPSS and RL-SAGE methods identified many novel sense and antisense transcripts, which are differentially expressed at the two important growth stages of *M. grisea*. The identified novel transcripts, especially those specifically expressed in appressoria, are valuable genomic resource for a better understanding of the molecular basis of *M. grisea *pathogenicity. The established MPSS and RL-SAGE websites provide useful genomics resource for the public. The microarray (GSM126989) and RL-SAGE (GSM127012) data were deposited at the NCBI-GEO website and also the MPSS data can be downloaded at the *M. grisea *MPSS website [42].

## Methods

### Fungal strains, growth conditions and RNA isolation

The *M. grisea *strain 70–15 was chosen for the transcriptome profiling because of the availability of its whole genome sequence [13]. The mycelia of 70–15 was cultured on a liquid medium [0.2% (w/v) yeast extract and 1% (w/v) sucrose] for 72 h (28°C at 200 rpm). The harvested mycelia were filtered and grinded for RNA isolation using the TRIzol method (Invitrogen, CA). For isolation of total RNA from the germinating appressoria, the mycelia of 70–15 were grown for two weeks on oatmeal agar plates and then the conidia were induced under white fluorescence light for five days. About 2 ml of conidia suspension (5 × 10^5 ^spores ml^-1^) was sprayed on Falcon Petri plates (150 mm × 15 mm) (Falcon, NJ) and the lids were covered with moist filter papers. These plates were incubated at 28°C and appressorium formation was monitored under a microscope at 6 h intervals. Fungal tissue was harvested 24 h after incubation since over 90% of the conidia extended a germ tube from the basal and/or the apical cell. Using a sterile blade, appressoria were scrapped and transferred quickly to the TRIzol solution, and then the suspension was centrifuged at 12,000 × g at 5°C for 5 min. About 2 g of appressorium pellet was collected and subjected for total RNA isolation. The poly (A^+^) mRNA was isolated from the total RNA using the Oligotex mRNA midi kit (Qiagen, CA).

### MPSS library construction and sequence analysis

MPSS library construction was carried out at Solexa, Inc. (Hayward, CA) as described by Brenner et al [6] and Meyers et al [7]. About 500 μg of total RNA isolated from mycelium and appressorium tissues (described above) were used in the MPSS library construction. The entire data set is available at [42]. All tags were normalized to tags per million (TPM) as described by Meyers et al [7].

### RL-SAGE library construction and sequence analysis

About 50 ng of mRNA isolated from mycelium tissue was used for RL-SAGE library construction as described by Gowda et al [5]. A total of 7,292 sequence reads from the RL-SAGE library were sequenced at Arizona Genomics Institute. The ditags and distinct tags were extracted from these sequences using SAGEspy program developed at the Ohio Supercomputer Center [43]. All RL-SAGE tags from mycelium library are available from the MGOS database [44].

### Annotation of MPSS and RL-SAGE tags

The distinct MPSS and RL-SAGE tag sequences were matched to the *M. grisea *reference sequences including the genomic DNA, annotated genes (CDS), and 500 bp upstream (putative 5'UTR) and downstream (putative 3'UTR) regions that are available from the Broad Institute (version 5.0, release in January, 2006 [15]). The EST dataset of *M. grisea *from the TIGR database was used for matching MPSS and RL-SAGE tags release 5.0 on April 28, 2004 [16]). We also used a number of tools developed at the Ohio Supercomputer Center [43], the *Magnaporthe grisea Oryza sativa *(MGOS) database [44] and the University of Delaware MPSS database [42]for data analyses. We identified the antisense transcripts from MPSS and RL-SAGE tags by converting all of the tags to antisense orientation using a reverse-complementation procedure before matching to the various sequences of *M. grisea*.

### The *M. grisea *oligoarray and the hybridization procedures

The *M. grisea *oliogoarray chip containing 60-mer oligos representing a total of 21,885 probes was obtained from Agilent (G4137A; Wilmington, Delaware). Of the 21,885 probes, 13,666 are from the annotated genes of *M. grisea *and 7,144 probes are from the rice ESTs [18]. The remaining 1,075 probes include quality controls, positive controls and negative controls. The total RNA was isolated from mycelia and appressoria using the TRIzol reagent (Invitrogen) following the manufacturers suggested protocol. Prior to hybridizations, quality and quantity of the total RNA sample was confirmed by running an agarose gel electrophoresis and by using a spectrophotometer. In the oligoarray hybridization experiments, we included six technical replicates of one RNA sample from mycelial and appressorial tissue, of which three were dye-reversal. About 500 ng of total RNA was used as template for cRNA production, and Cyanine dyes were incorporated using the Agilent low RNA input linear amp kit (5198-3523; Agilent). Normal yields from 500 ng total RNA input using an in vitro transcription were 15 μg cRNA (15 pmole cyanine dye incorporated/ug cRNA). One μg of labeled cRNA (Cy3 and Cy5 labeled sample) was diluted to 175 μl and defragmented at 60°C for 30 min following the Agilent hybridization protocols (5184-3568; Agilent). Defragmented samples were diluted to 500 μl (30% formamide final concentration) and hybridized for 20 h at 40°C. Arrays were washed, dried and scanned on an Agilent G2565BA microarray scanner described by [9]. The raw TIFF images were analyzed using the Agilent Feature Extraction software v 8.1 using the recommended default settings.

### Microarray data and KOG analysis

To minimize the variation in probe labeling and detection, intensities of Cy3- and Cy5-labeled probes were normalized using subgrid LOWESS normalization. Spots with lower signal intensity than the negative controls or with intensities less than twice the average background for the channel were manually blocked (flagged) from further analysis. This corrected and normalized dye bias data eliminated larger component of the variance, thus giving a greater confidence to the evaluated (treatment) and reference (control) data for testing the statistical significance. The genes with valid signals in all six replicates were exported to Partek Pro v.6.0 software (Partek Inc., Missouri). The normalized values were used to calculate the ratio of channel intensities (Cy5/Cy3), which were then log_2 _transformed. The transformed ratio was plotted in a histogram with ± two standard deviations away from the mean. A ± 1.7-fold increase or decrease in signal intensity or ± 0.77 on the Log_2 _scale from the histogram was considered to indicate genes that are differentially expressed. The normalized data were then subjected to ANOVA model using Boenferoni method [45,46]. FDR (≤ 0.05) [47,48] was calculated based on the *p*-value (≤ 0.001) from ANOVA. This stringent criterion limits the ability of the oligoarray experiment to detect small but biologically important changes between the appressorium and mycelium at approximately a 95% confidence interval. The genes that are significantly and differentially expressed in the appressorial and mycelial oligoarray data were used to compare the appressorial MPSS data with mycelial MPSS and RL SAGE data. Appressorium and mycelium-differentially expressed genes from the microarray analysis were functionally categorized using the euKaryotic Orthologous Groups (KOGs) database [19]. The gene sequences were blasted against the KOG database with E-value of 1.0e-20.

## Authors' contributions

MG designed the study, performed the experiments, constructed RL-SAGE library, conducted data mining, and wrote the article. RCV performed MPSS, RL-SAGE, and oligoarray data analysis and assisted in writing the article, MBR performed oligoarray data analysis and revised the article, KN constructed the *M. grisea *MPSS database, HL and ES designed MPSS and RL-SAGE tag analysis software, HRK and RW sequenced the RL-SAGE libraries, SC performed oligoarray experiment, CDH performed MPSS library construction, RBD assisted in RL-SAGE clone sequencing, BHN performed KOG analysis, BM and GLW contributed to conception and design of the study and revising and writing the article. All authors read and approved the manuscript.

## Supplementary Material

Additional file 1 Appressorial and mycelial MPSS tags, microarray up/down regulated genes and KOG analysis results Click here for file

Additional file 2 Novel appressorial and mycelial MPSS and RL-SAGE tags. Click here for file

Additional file 3 Alternative sense and antisense transcript tags for selected genes. Click here for file

Additional file 4 Antisense transcript tags for annotated genes. Click here for file

Additional file 5 Annotated genes with sense and antisense tags specifically and commonly present in mycelia and appressoria. Click here for file

Additional file 6 List of alternative transcript tags from MPSS and EST. Click here for file

Additional file 7 Alternative tags for selected genes in appressoria and mycelia identified by MPSS. Click here for file

Additional file 8 Common alternative tags detected by RL-SAGE and MPSS. Click here for file

Additional file 9 List of top 20 highly and specifically up regulated and down regulated genes in appressoria and in mycelia of *M. grisea *detected by oligoarray and MPSS. Click here for file

Additional file 10 The up-regulated genes (4,649) in appressoria identified by oligoarray analysis were subjected for KOGs analysis based on the putative function in the KOGs protein database. Functional classification and percentage of genes represented in appressorial tissue is shown. Click here for file

Additional file 11 The up-regulated genes (3,784) in mycelia identified by oligoarray analysis were subjected for KOGs analysis based on the putative function in the KOGs protein database. Functional classification and percentage of genes represented in mycelia tissue is shown. Click here for file

## Figures and Tables

**Table 1:** MPSS and RL-SAGE libraries from mycelial and appressorial stages of 70-15 strain of *M. grisea*

Library	MPSS		RL-SAGE
Tissue	Appressoria	Mycelia	Mycelia
Growth stage	24-hr after germination	96-hr liquid culture	96-hr liquid culture
Anchoring enzyme and tag size	GATC (*Dpn*II) + 13 bases	GATC (*Dpn*II) + 13 bases	CATG (*Nla*III) + 17 bases
No. of genomic virtual tags ^1^	286,874	286,874	355,837
No. of total tags per library	1,459,146	1,326,720	245,873
No. of unique tags per library	16,427	20,299	51,925
No. of copies per tag	2 to 28,845	1 to 30,910	1 to 7,383
No. of significant tags ^2^	12,927	12,531	16,580
No. of non-significant tags ^3^	3,500	7,768	35,345
No. of significant antisense tags ^4^	653 (5.1%)	1,825 (14.6%)	605 (3.6%)

^1^Genomic tags were extracted from the *M. grisea *draft genomic sequence as described in the text.

^2^Significant tags are those with ≥ 2 copies per tag in RL-SAGE and ≥ 4 copies per tag in MPSS.

^3^Non-significant tags are those with one copy per tag in RL-SAGE and ≤ 3 copies per tag in MPSS.

^4^Significant antisense tags are those with ≥ 2 copies per tag in RL-SAGE and ≥ 4 copies per tag in MPSS matched to the annotated genes

**Table 2:** Matching analysis of MPSS and RL-SAGE tags to *M. grisea *EST and genomic sequences.

MgRefSeq^1^	Library	MPSS				RL-SAGE	
		
		Appressoria		Mycelia		Mycelia	
		Significant tags (%)	Non Significant tags (%)	Significant tags (%)	Non Significant tags (%)	Significant tags (%)	Non Significant tags (%)

Genomic DNA	Both sense + antisense	10,957 (84.8)	2,335 (66.7)	10,649 (85.0)	5,912 (76.1)	10,772 (65.0)	8,950 (25.3)
ESTs	Sense	5,597 (43.3)	753 (21.5)	5,557 (44.3%)	1,980 (25.5)	3,684 (22.2)	2,218 (6.3)
	Antisense	1,185 (9.2)	415 (11.8)	2,374 (18.9)	1,912 (24.6)	1,572 (9.5%)	1,419 (4.0)
	Both sense + antisense	6,620(51.2)	1,149 (32.8)	7,750 (61.8)	3,842(49.5)	5,067 (30.6)	3,567 (10.0)
Coding region (CDS)	Sense	4,574 (35.4)	690 (19.7)	4,761 (38.0)	1,935 (24.9)	2,058 (12.4)	1,751 (5.0)
	Antisense	653 (5.1)	329 (9.4)	1,825 (14.6)	1,837 (23.6)	605 (3.6)	890 (2.5)
	Both sense + antisense	5,205 (40.3)	1,016 (29.0)	6,560 (52.4)	3,759 (48.4)	2,648 (16.0)	2,638 (7.5)
500 bp upstream sequences^2^	Sense	1,730 (13.4)	367 (10.5)	1,352 (10.8)	715 (9.2)	2,548 (7.2)	1,399 (4.0)
	Antisense	699 (5.4)	284 (8.1)	906 (7.2)	655 (8.4)	2,495 (7.0)	1,204 (3.4)
	Both sense + antisense	905 (7.0)	301 (8.6)	877 (7.0)	630 (8.1)	964 (5.8)	962 (2.7)
500 bp downstream sequences^3^	Sense	4,075 (31.5)	618 (17.7)	2,986 (23.8)	1,097 (14.1)	3,117 (6.0)	4,842 (29.2)
	Antisense	3,687 (28.5)	581 (16.6)	3,044 (24.3)	1,037 (13.4)	2,251 (4.3)	3,997 (24.1)
	Both sense + antisense	4,189(32.4)	600 (17.1)	3,065 (24.5)	1,005 (12.9)	5,131 (30.9)	2,799 (7.9)
Number of genes transcribed^4^	-	7,686	2,115	6,735	4,234	6,029	5,163
Novel transcript tags-TIGR ESTs^5^	-	4,478 (34.64)	1,220 (34.85)	3,297 (26.3)	2,281 (29.4)	5,887 (35.5)	5,499 (15.55)
Novel transcript tags-COGEME ESTs^6^	-	7,573 (58.6)	1,876 (53.6)	6,737 (53.8)	4,607 (59.3)	7,366 (44.4)	7,167 (20.3)
Putative intergenic tags^7^	-	1,618 (12.5)	571 (16.3)	1,211 (9.7)	977 (12.6)	2,716 (16.4)	3,004 (8.5)
Putative intergenic tags match ESTs^8^	-	394 (3.0)	70 (2.0)	395 (3.1)	189 (2.4)	596 (3.6)	437 (1.2)

^1 ^*Magnaporthe grisea *reference sequences.

^2 ^Probably 5'UTR region.

^3 ^Probably 3'UTR region.

^4^Number of genes (including single and multiple hits) expressed using CDS, 500 bp upstream and downstream sequences.

^5^Tags matched to the genome sequence but not to TIGR-*M. grisea *ESTs.

^6^Tags match to the genome sequence but not to COGEME *M. grisea *ESTs.

^7^Tags matched to the genomic sequence but not to the CDS, 500 bp upstream or downstream sequences.

^8^Putative intergenic tags that match to *M. grisea *ESTs deposited either in TIGR or COGEME

**Table 3:** Alternative sense and antisense ESTs, MPSS tags and RL-SAGE tags for *M. grisea *genes.

Annotated M. grisea gene		EST		RL-SAGE		MPSS	
Gene ID	Putataive function	Sense tag*	Antisense tag*	Sense tag: CATG+17 bases (No. of tags in mycelia)	Antisense tag: CATG+17 bases (No. of tags in mycelia)	Sense tag: GATC+17 bases (No. of tags in appressoria:mycelia)	Antisense tag: GATC+17 bases (No. of tags in appressoria:mycelia)
MGG_07177.5	-	Mg2_0141_01	mgmt016xN19.f	ACAAGACCTGCCCCAAG (1)	TTTGCTAGTTTGTTTTG (3)	ACGCATATCATGC (52:0)	-
		Mg2_2405_01				TCAGACTTTCGGT (303:0)	
MGG_12892.5	Ribosomal protein, S9	Mg2_7114_01	mgns015xC05.f	GACATCCGTGTCCGCGT (7)	ATTGCGGTGTCTGTGGA (6)	GTGGCCTACTACC (15:0)	GACTTGGCGATAG (0:10)
				TCGTCAAACAAGCAGAG (3)		CATCACTTGCTCC (24:0)	
						GAATTTCCTGGCC (138:0)	
						TTGAGCAATGCTT (16:58)	
MGG_05296.5	Ribosomal protein, L34	Mg2_2151_01	mgmt013xN14.f	TCCTGTGACGAACGACG (16)	CAGACAAGAACGCCTTT (34)	TCTTTATCGGAAC (3240:0)	TGAGCGTACTCGC (0:11)
				ATAGAGAAGACCAAAAT (167)		GAGGAGCAGAAGA (44:0)	
						GATGACGGCCAAC (7:0)	
						TCCAAGCCCAAGA (0:10)	
MGG_12894.5	DEAD box protein	Mg2_0648_01	mgmk001xO10.f	GACTAAACAGGCCTCAT (60)	GGCAGGTCGTTGGCACC (1)	CGTCTCACGGTGA (516:740)	CCGTCTCAGCGAT (0:6)
		Mg2_2718_01		TAGTTCACACTGGAGAC (6)	GTGGCGACCGCCGCGGC (1)	TACTTGGCGTCCT (0:7)	
		Mg2_7473_01		GTTGCCACCGACGTTGC (1)	GCCTCGCAAGGCCTTAG (1)	CACGGTGACAAGC (15:16)	
				TCTAACCTAGGCGCAGG (1)	GCCATTCAAGGGCCGGT (10)	AGGCACATGGAGA (0:8)	
MGG_12958.5	MST12 – transcription factor	Mg2_2683_01	mgmk004xA02.f	ATGTGAGCTTGCTTATT (9)	-	AACCAGGGTCACC (0:5)	-
		Mg2_3285_01				CCGACCTGTGGTC (22:6)	
						AGACAACCTAGCA (66:106)	
						ACAGAAAACCTTT (0:315)	
MGG_07752.5	ATP synthase alpha chain-like protein	Mg2_2666_01	mgmk015xO19.f	AAGCAGGTCGCTGGTTC (27)	GCCTTGAGCTGGGCAGC (1)	CTGCAATGGGAGG (4041:1459)	CTTTGCTCGAGAA (0:6)
		Mg2_2707_01	mgmk019xB20.f	CGTTATTCGGTGGTTGT (3)		GGGGAATGGTTCC (0:6)	GTCGAAGATGACG (0:6)
			mgmk018xA01.f			TGTCCAAGCAGGC (0:13)	
						TTCGCTGGTGTCA (19:10)	

* EST data obtained from the MGOS database [44]

**Table 4:** Frequency of the alternatively transcribed sense and antisense tags for the coding regions of *M. grisea*.

	Sense tags				Antisense tags		
	MPSS		RL-SAGE		MPSS		RL-SAGE

No. of alternative tags	Appressoria	Mycelia	Mycelia	No of antisense tags/gene	Appressoria	Mycelia	Mycelia

≥ 5	28	124	46	≥ 5	1	111	14
4	26	117	76	4	1	90	23
3	168	394	197	3	57	191	108
2	715	992	636	2	173	455	293
No of genes with ≥ 2 tags (%)	937 (20.8)	1,631 (35.0)	955 (27.5)	No of genes with ≥ 2 tags (%)	232 (19.9)	847 (31.8)	438 (24.0)
No of genes with single tag (%)	3,566 (79.2)	3,028 (65.0)	2,518 (72.5)	No of genes with single tag (%)	933 (80.1)	1,809 (68.2)	1,389 (76.0)

**Table 5:** Comparison of *M. grisea *transcriptomes measured by MPSS, RL-SAGE and oligoarray approaches.

Technology Comparison	Correlation coefficient using all tags	Correlation coefficient using only significant tags	Correlation coefficient using tags ≥ 10 copies	Correlation coefficient using tags ≥ 25 copies
Appressoria: MPSS vs microarray ^1^	0.59	0.60	0.65	0.69

Appressoria: MPSS vs microarray ^2^	0.67	0.70	0.74	0.77
Mycelia: MPSS vs microarray ^1^	0.17	0.16	0.18	0.16
Mycelia: MPSS vs microarray ^3^	0.49	0.49	0.51	0.51
Mycelia: RL-SAGE vs microarray ^1^	0.2	0.19	0.29	-0.29
Mycelia: RL-SAGE vs microarray ^4^	0.31	0.33	0.64	0.43
Mycelia: MPSS vs RL-SAGE ^1^	0.068	NA	NA	NA
Mycelia: MPSS vs RL-SAGE ^5^	0.4	NA	NA	NA

^1^Correlation before removing genes.

^2^Correlation after removing 4 genes out of 1729 genes (Standard residuals range ≤ -5.7 to ≥ 9.4).

^3^Correlation after removing 10 genes out of 914 genes (Standard residuals range ≤ -1.75 to ≥ 5.3).

^4 ^Correlation after removing 14 genes out of 883 genes (Standard residuals range ≤ -1.75 to ≥ 5.3).

^5 ^Correlation after removing 18 genes out of 276 genes (Standard residuals range ≤ -0.45 to ≥ 2.8). NA-Not available.
