# Supplementary material for: Deep and comparative analysis of the mycelium and appressorium transcriptomes of Magnaporthe grisea using MPSS, RL-SAGE, and oligoarray methods
Source: BMC Genomics. 2006 Dec 8;7:310. doi: 10.1186/1471-2164-7-310 (PMC1764740; doi:10.1186/1471-2164-7-310)
Supplement: Additional file 3 — Alternative sense and antisense transcript tags for selected genes. [file 1471-2164-7-310-S3.doc]

**Additional File 3. Alternative tags for selected sense and antisense transcripts**

|  |  | Mycelia - RL-SAGE | | Mycelia - MPSS | | Appressoria - MPSS | |
| --- | --- | --- | --- | --- | --- | --- | --- |
| Genes | Locus ID/NCBI accession # | Sense tags | antisense tags | sense tags | antisense tags | sense tags | antisense tags |
| HSP70 | MGG_02503.5 | 4* | 1 | 11 | 8 | 2 | 2 |
| Hydrophobin (MPG1) | L20685 | 1 | 0 | 2 | 0 | 2 | 1 |
| 60S ribosomal protein L17 | MGG_09194.5 | 2 | 2 | 0 | 0 | 0 | 0 |
| Vacuolar ATP synthase subunit B | MGG_03244.5 | 4 | 2 | 2 | 2 | 1 | 0 |
| Calmodulin | MGG_06884.5) | 4 | 3 | 1 | 1 | 1 | 1 |
| Phosphoglycerate kinase | MGG_05063.5 | 4 | 2 | 2 | 1 | 1 | 0 |
| Histidine kinase | MGG_11174 | 2 | 2 | 0 | 1 | 0 | 0 |
| Polyubiquitin | MGG_01282.5 | 2 | 2 | 6 | 3 | 5 | 1 |
| Glyceraldehydes 3-phosphate dehydrogenase | MGG_01084.5 | 4 | 3 | 2 | 2 | 2 | 0 |

* Numbers indicate the total number of different RL-SAGE or MPSS tags matched to each gene.
