# Supplementary material for: Deep and comparative analysis of the mycelium and appressorium transcriptomes of Magnaporthe grisea using MPSS, RL-SAGE, and oligoarray methods
Source: BMC Genomics. 2006 Dec 8;7:310. doi: 10.1186/1471-2164-7-310 (PMC1764740; doi:10.1186/1471-2164-7-310)
Supplement: Additional file 7 — Alternative tags for selected genes in appressoria and mycelia identified by MPSS. [file 1471-2164-7-310-S7.doc]

Additional File 7. Alternative tags of known genes in appressoria and mycelia identified by MPSS

| MPSS Tag Sequence | Appressoria  (Copy Number) | Mycelia  (Copy Number) | Gene I.D. | Gene Annotation |
| --- | --- | --- | --- | --- |
| GATCCGCGGTGTCCAGG | 2 | 0 | MGG_07752.5 | Mitochondrial F1 ATPase subunit alpha |
| GATCTTCGCTGGTGTCA | 25 | 0 |
| GATCCTGCAATGGGAGG | 4761 | 1528 |
| GATCGATGCACCAGTGA | 2 | 0 |
| GATCGATGACTGCTCTG | 8 | 0 |
| GATCTTGGCGACGGGGA | 2 | 0 |
| GATCCTCCTCCGTGCGT | 0 | 2 |
| GATCTGTCCAAGCAGGC | 0 | 13 |
| GATCTTCTTGGAGGCCG | 0 | 13 |
| GATCGTCGAAGATGACG | 0 | 6 |
| GATCTTCGCTGGTGTCA | 0 | 14 |
| GATCCTTTGCTCGAGAA | 0 | 6 |
| GATCATTGAGACCCAGG | 0 | 2 |
| GATCGGGGAATGGTTCC | 0 | 6 |
| GATCTTGGCGACGGGGA | 0 | 4 |
| GATCTCAAGAAGAAGCG | 213 | 0 | MGG_03982.5 | Actin |
| GATCTGTACAACAACAT | 11 | 3 |
| GATCTTGACCTTCATCG | 2 | 4 |
| GATCTCGAAGCAGGAGT | 2825 | 788 |
| GATCGGTGGTTCCATTC | 50 | 10 |
| GATCCACATCTGCTGGA | 0 | 3 |
| GATCATGAAGTGCGATG | 0 | 2 |
| GATCCAGACTGCTGCCC | 0 | 1 |
| GATCCAGACGGAGTACT | 0 | 7 |
| GATCTGCGTCATCTTCT | 0 | 5 |
| GATCATTGCTCCCCCCG | 0 | 4 |
| GATCACTGCCCTGGCAC | 0 | 8 |
| GATCAGAGGCAGAGTGG | 11 | 2 | MGG_03201.5 | Hypothetical protein similar to acetyl-coenzyme A synthase |
| GATCCACTGTTCCTTCT | 9 | 0 |
| GATCCTCGCCGGCGAGG | 14 | 0 |
| GATCCACGGCAATGACG | 50 | 0 |
| GATCGTTGGAAAGGAAC | 7 | 0 |
| GATCCAATAAAACCCTT | 0 | 17 |
| GATCCGAGCAGCGGGCG | 0 | 7 |
| GATCCTCGCCGGCGAGG | 0 | 1 |
| GATCCTTGAGCGCATGC | 0 | 37 |
| GATCATCCGCCGTCTGG | 11 | 0 | MGG_06609.5 | Acetyl-CoA hydrolase |
| GATCCTGGAGGCTTACT | 1410 | 279 |
| GATCCAGGGCGTGGAGG | 6 | 0 |
| GATCCGAAGTCCACCGA | 0 | 3 |
| GATCAACTTTTTCGACA | 0 | 4 |
| GATCGAGTACTTTGAGC | 0 | 3 |
| GATCAAACGTTGTTGGT | 17 | 15 | MGG_01282.5 | Ubiquitin |
| GATCTTCGCTGGAAAGC | 119 | 29 |
| GATCTTCGTGAAGACTC | 11 | 6 |
| GATCTTTGCGGGCAAGC | 4658 | 3624 |
| GATCCAGGACAAGGAGG | 8 | 38 |
| GATCAGTCGCTGTTGGT | 0 | 4 |
| GATCCAAGACAAGGAGG | 0 | 7 |
| GATCTTCGACTTTACAT | 0 | 21 |
| GATCGTCAGCAAGGGCG | 5 | 0 | MGG_03670.5 | Conserved hypothetical protein |
| GATCCACCAGGCTGTTG | 6472 | 2428 |
| GATCCAGAAAGTCCACG | 14 | 11 |
| GATCGGCAGCAAGACCG | 0 | 2 |
| GATCAGCCTTGAGCTTC | 0 | 4 |
| GATCCTGTCATCTTCCA | 0 | 12 |
| GATCCAGGTGGACTGGA | 0 | 17 |
| GATCGGGGCAGCGCCGT | 0 | 3 |
| GATCTCAGAGAACTTGC | 17 | 233 | MGG_03641.5 | Elongation factor 1-alpha |
| GATCGGTGGTATCGGCA | 26 | 267 |
| GATCCTTGAGAAGCTTG | 1013 | 1791 |
| GATCTCGGAGTCCGCCG | 18 | 36 |
| GATCTACAAGCTGAAGG | 0 | 22 |
| GATCGAGAAGTACGAGA | 0 | 14 |
| GATCTTGTAGACATCCT | 0 | 29 |
| GATCATGTGGTCACCGT | 0 | 24 |
| GATCTGCTTGAAGGCGT | 23 | 46 | MGG_06656.5 | ADP,ATP carrier protein |
| GATCCGCGCCGGCCGTC | 6 | 0 |
| GATCTACGACCAGCTCC | 23 | 163 |
| GATCGTCGCCAAGGAGG | 87 | 110 |
| GATCGACAGCACACCAG | 0 | 6 |
| GATCCACCCTTGAAGGC | 0 | 20 |
| GATCATCTCCTCCTGGT | 0 | 6 |
