# Supplementary figures and images for: Deep and comparative analysis of the mycelium and appressorium transcriptomes of Magnaporthe grisea using MPSS, RL-SAGE, and oligoarray methods
Source: BMC Genomics. 2006 Dec 8;7:310. doi: 10.1186/1471-2164-7-310 (PMC1764740; doi:10.1186/1471-2164-7-310)

## Slide 1
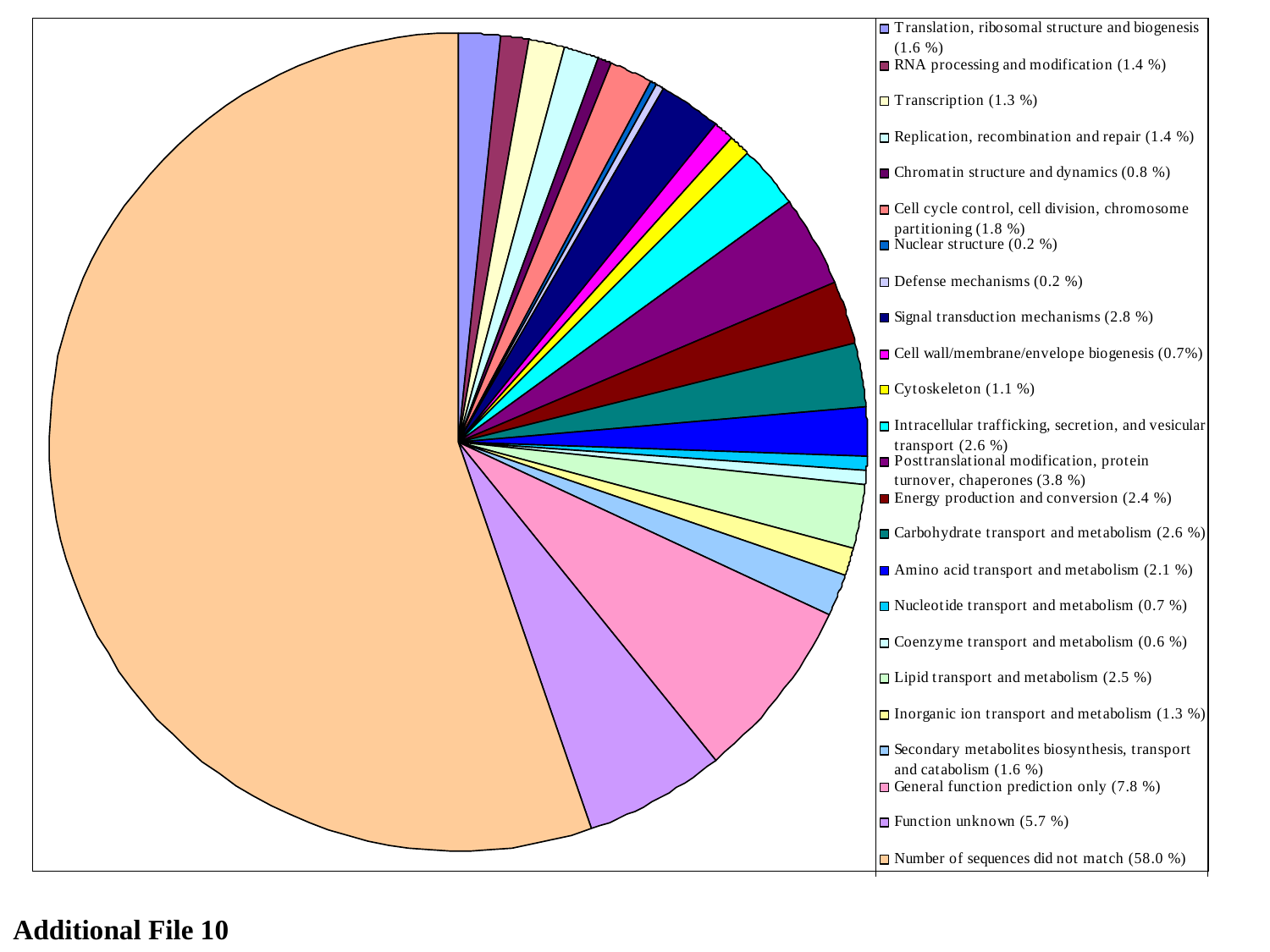

Additional File 10

Supplement: Additional file 10 — The up-regulated genes (4,649) in appressoria identified by oligoarray analysis were subjected for KOGs analysis based on the putative function in the KOGs protein database. Functional classification and percentage of genes represented in appressorial tissue is shown. [file 1471-2164-7-310-S10.ppt]

## Slide 1
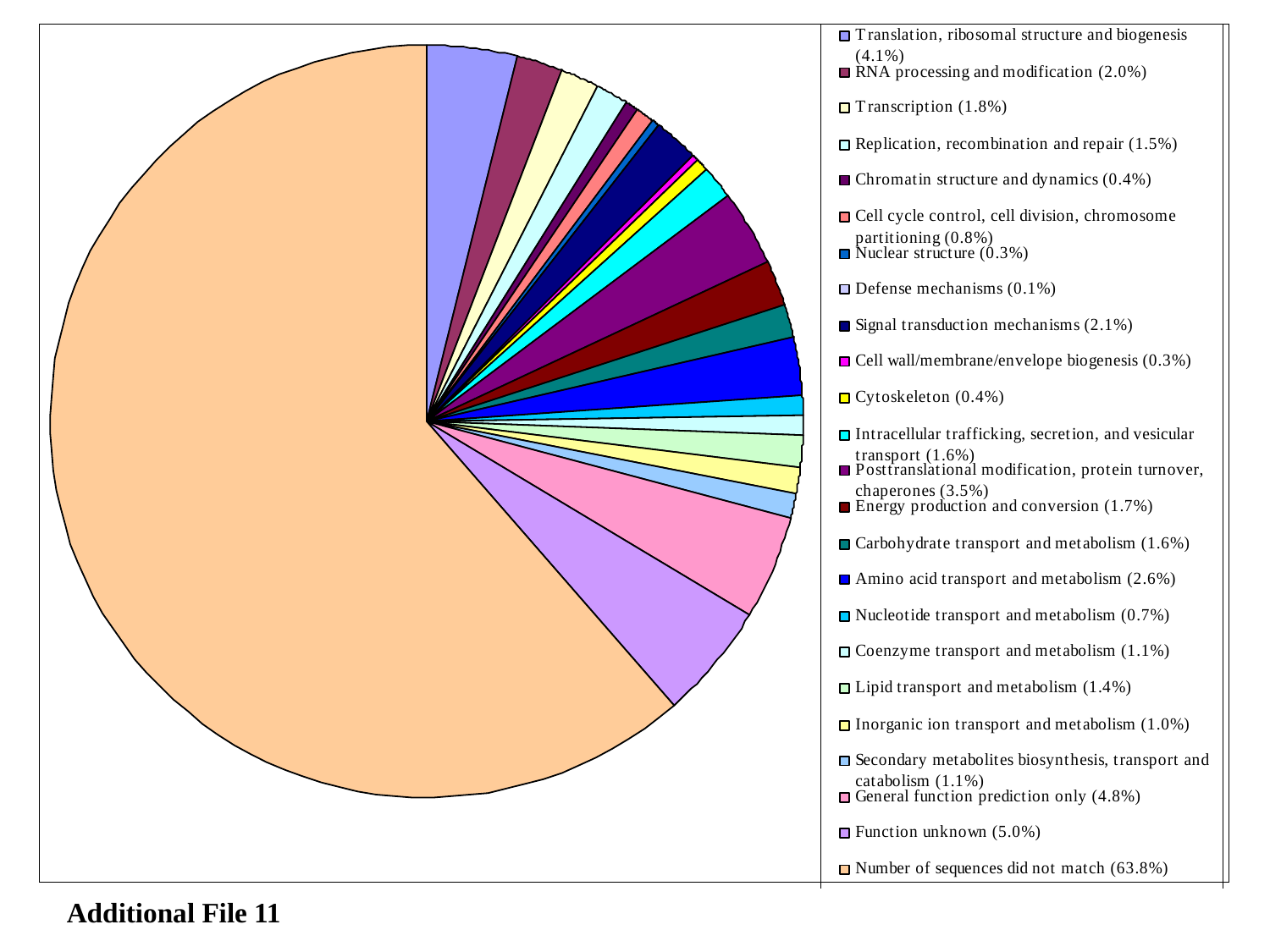

Additional File 11

Supplement: Additional file 11 — The up-regulated genes (3,784) in mycelia identified by oligoarray analysis were subjected for KOGs analysis based on the putative function in the KOGs protein database. Functional classification and percentage of genes represented in mycelia tissue is shown. [file 1471-2164-7-310-S11.ppt]
